# Genome-wide profiling of gene expression in the epididymis of alpha-chlorohydrin-induced infertile rats using an oligonucleotide microarray

**DOI:** 10.1186/1477-7827-8-37

**Published:** 2010-04-22

**Authors:** Shuwu Xie, Yan Zhu, Li Ma, Yingying Lu, Jieyun Zhou, Youlun Gui, Lin Cao

**Affiliations:** 1 Department of Reproductive Pharmacology, Shanghai Institute of Planned Parenthood Research, Shanghai 200032, China; 2 National Population and Family Planning Key Laboratory of Contraceptive Drugs and Devices, Shanghai 200030, China; 3 Department of Cancer and Cell Biology, the Vontz Center for Molecular Study, University of Cincinnati, 3125 Eden Avenue, Cincinnati, OH, USA; 4 Obstetrics and Gynecology Hospital of Fudan University, Shanghai, 200011, China

## Abstract

**Background:**

As one of the chlorinated antifertility compounds, alpha-chlorohydrin (ACH) can inhibit glyceraldehyde-3-phosphate dehydrogenase (G3PDH) activity in epididymal sperm and affect sperm energy metabolism, maturation and fertilization, eventually leading to male infertility. Further studies demonstrated that the inhibitory effect of ACH on G3PDH is not only confined to epididymal sperm but also to the epididymis. Moreover, little investigation on gene expression changes in the epididymis after ACH treatment has been conducted. Therefore, gene expression studies may indicate new epididymal targets related to sperm maturation and fertility through the analysis of ACH-treated infertile animals.

**Methods:**

Rats were treated with ACH for ten consecutive days, and then each male rat copulated with two female rats in proestrus. Then sperm maturation and other fertility parameters were analyzed. Furthermore, we identified epididymal-specific genes that are associated with fertility between control and ACH groups using an Affymetrix Rat 230 2.0 oligo-microarray. Finally, we performed RT-PCR analysis for several differentially expressed genes to validate the alteration in gene expression observed by oligonucleotide microarray.

**Results:**

Among all the differentially expressed genes, we analyzed and screened the down-regulated genes associated with metabolism processes, which are considered the major targets of ACH action. Simultaneously, the genes that were up-regulated by chlorohydrin were detected. The genes that negatively regulate sperm maturation and fertility include apoptosis and immune-related genes and have not been reported previously. The overall results of PCR analysis for selected genes were consistent with the array data.

**Conclusions:**

In this study, we have described the genome-wide profiles of gene expression in the epididymides of infertile rats induced by ACH, which could become potential epididymal specific targets for male contraception and infertility treatment.

## Background

Although sperm are initially produced in the testes of mammals, they are incapable of capacitation and fertilization. Spermatozoa become mature and acquire fertilizing capacity during the passage through the epididymis [1,2]. During the process of sperm maturation in the epididymis, multiple changes occur in the sperm, including changes in morphology, biochemistry, physiology and the acquisition of fertilizing ability due to the interaction of epididymal secretory proteins with the spermatozoa [3-5]. Some processes of epididymal sperm maturation, such as substance metabolism and the initiation of progressive motility, can be selectively interrupted, which induces dysfunction of sperm fertilization and male infertility [6]. Additionally, disrupting epididymal sperm maturation does not interfere with testicular endocrine output and sperm production or affect testosterone generation and male libido [7,8]. Therefore, the process of sperm maturation in the epididymis may be an advantageous post-testicular target for the development of safe, rapid and reversible male contraceptives [9].

Further studies displayed that the inhibitory effect of ACH on G3PDH is not only confined to epididymal sperm but also to the epididymis [10]. Other findings suggested that ACH can affect epididymal function through multiple pathways, including inhibiting androgen dependent enzymes such as ATPase and AChE in the epididymis [11], influencing some markers involved in epididymal function such as glucosidase activity, acid and alkaline phosphatase activity, and sialidase activity [12-14], regulating the epididymal microenvironment such as acidity, fluid resorption and salt metabolism [15,16], and interfering with sugar transport, lipid metabolism and epididymal protein secretion [17-19]. All of the above effects on epididymal function through ACH indicate that it may influence male fertility by interfering with the epididymal milieu, in which the spermatozoa mature, rather than directly affecting spermatozoa. Moreover, little investigation on gene expression changes in the epididymis after ACH treatment has been conducted. Therefore, gene expression studies may indicate new epididymal targets related to sperm maturation and fertility through the analysis of ACH-treated infertile animals.

Some advances in researching epididymal-specific gene expression and function have been achieved. Transgenic technologies have generated temporally and spatially restricted targeted gene disruptions, which provide promise for our progress in understanding epididymal function and sperm maturation [20]. Gene silencing agents, such as RNAi, can manipulate gene expression and have been proven to be useful for the analysis of epididymal genes involved in sperm maturation and fertility [21]. Microarray technology has been widely used for the simultaneous examination of the expression of multiple genes and gene families for more than a decade. Microarray techniques are advantageous for gene expression assays because they have high sensitivity, permit analysis with a smaller amount of cells or tissues, and allow simultaneous analysis of a wide range of genes. In this present study, taking advantage of an oligonucleotide microarray, we evaluated the effects of ACH on gene expression in the epididymis, identified new genes related to epididymal function that possibly affect sperm maturation and male fertility, and provided some novel epididymal targets for male contraception and infertility research.

## Methods

### Animals and treatment procedure

Adult Sprague-Dawley rats were obtained from Sino-British Sippr/BK Lab Animal Co. Ltd. (Shanghai, China), maintained under controlled light (12L: 12D) and temperature (23°C), and provided with food and water ad libitum. Male rats (330-350 g) were randomly divided into two groups and gavaged with 1 ml/kg of solvent (without any ACH, control) or 10 mg/kg ACH (Sigma Chemical Co., St. Louis, MO. FW: 110.5) (treated) suspended in a 0.5% methylcellulose solution containing 0.2% Tween 20 for 10 consecutive days. Virgin female rats (220-250 g) were used for the mating study. At sacrifice, animals were anesthetized, and blood was collected from the abdominal aorta for testosterone (T) and dihydrotestosterone (DHT) assays; then the testes, seminal vesicles, ventral prostates and epididymides were weighed. The left epididymides were immediately frozen in liquid nitrogen for RNA extraction, and the right epididymides were used for analysis of sperm morphology and motility. All experimental studies were approved by the Shanghai Experimental Animal Ethics Committee and complied with Regulations on the Care and Use of Laboratory Animals promulgated by The Ministry of Science and Technology of China.

### Serumal T and DHT array

The serum levels of T and DHT in rats were detected by an enzyme linked immunosorbent assay using a T and DHT Elisa kit (Adlitteram diagnostic laboratories, USA) and were analyzed with a microplate and cuvette spectrophotometer Zenyth 200 (Anthos Inc, Austria) according to the manufacturer's instructions.

### Sperm motility and morphology analysis

Sperm samples were collected from the distal cauda of the right epididymis and were used for computer-assisted sperm analysis (CASA) on the HTM-IVOS, (Hamilton-Thorne Research, Beverly, MA) using version 12 of the Toxicology Software. Approximately 5000 cauda epididymal sperm were analyzed for each treatment group (n = 8). The following kinematic parameters of motility were determined by CASA: average path velocity (VAP), curvilinear velocity (VCL), straight-line velocity (VSL), amplitude of lateral head displacement (ALH), beat cross frequency (BCF), linearity (LIN = VSL/VCL ×100) and straightness (STR = VSL/VAP × 100).

As for morphological analysis, the remaining sperm samples from the motility analysis were fixed with 10% neutral buffered formalin. Samples were aliquoted onto slides, and sperm were analyzed using the 10× phase contrast objective (Motic Group Co. Ltd., GD China). Approximately 200 to 300 sperm per sample were analyzed, and abnormal morphological parameters were broken sperm (head only, tail only, other breakages) and angulated sperm (bent at midpiece or looped). Then, the frequency of these abnormalities was calculated. Besides the phase contrast objective analysis of sperm morphology, spermatozoa from the cauda epididymis fixed with 2.5% glutaral and 1% osmic acid were also used for electron microscopic analysis (Philips CM120 electron microscope, Eindhoven, Netherlands). In order to analyze cytoplasmic droplet retention, approximately 80 to 100 midpiece sperm flagellum trans-sections per male rat were photographed and the number of sperm with droplets was calculated.

### Mating and fertility evaluation

On the last day of treatment, each male rat was caged together with two female rats in proestrus overnight. Female rats were examined the next morning for the presence of sperm in their vaginal smears; this was defined as day 0 of gestation for sperm-positive animals. Females were sacrificed and examined for pregnancy status on gestation day 13, and the effect of α-chlorohydrin treatment on fertility was verified. Finally, the following male reproductive indices were calculated: mating index (number of sperm positive females/number of pairings), pregnancy index (number of pregnancies/number of sperm positive females) and fertility index (number of pregnancies/number of pairings).

### Statistical Analysis

Data are presented as mean ± SEM. The data assessing the serum androgen level, genital organ weight, sperm motility and pregnancy outcome were analyzed for the difference between the control and α-chlorohydrin treatment groups by One-Way ANOVA. Reproductive indices were analyzed with the Fisher Probability Exact test. Probabilities of less than 0.05 were considered statistically significant.

### Total RNA preparation

The total RNA was isolated from the left epididymides using a TRIzol reagent (Invitrogen, Carlsbad, CA) and further purified using the RNeasy Mini Kit (Qiagen, Valencia, CA), according to manufacturer's instructions. The total RNA was quantitatively determined by the ratio of absorbance at 260/280 nm, and the quality was identified with denaturing agarose gel electrophoresis.

### Microarray and raw probe signal processing

Double-stranded cDNA was synthesized with the One-cycle cDNA Synthesis Kit (Affymetrix) and purified using the GeneChip Sample Cleanup Module (Affymetrix). The cDNA was used as a template for biotin-labeled cRNA in vitro transcription using the GeneChip IVT Labeling Kit (Affymetrix). After cleanup and quantitative detection, the purified biotinylated target cRNA was processed into short sequences by fragmentation. The hybridization cocktail was comprised of 15 μg of fragmented biotin-labeled cRNA with Oligo B2 incorporated and a eukaryotic hybridization control. Subsequently, 80 μL of hybridization cocktail was hybridized onto the test chips to detect the cRNA integrity and confirm the validity of the system. The 3'-5' ratio of GAPDH and actin should be no more than 3.0. Next, RAE 230 2.0 microarrays (Affymetrix) were directly loaded with 200 μL of hybridization solution and put into a Genechip Hybridization Oven 640 (Affymetrix) rotating at 60 rpm at 45°C for 16 h. After hybridization, the arrays were washed on a Genechip Fluidics Station 400 (Affymetrix) and scanned using the Genechip Scanner 3000 (Affymetrix), according to the manufacturer's protocols. Microarray images were visually inspected for quality, and the probes with low signal intensity and excessively noisy background were removed before further analysis. The signal values were determined using the GeneChip Operating System 1.2 (GCOS, Affymetrix).

For each array, all the original probe sets were normalized to a mean signal intensity value of 500. The default GCOS statistical values were used for all analyses. The expression of transcripts on the array were considered "present" or "absent" if their detection *p*-values were lower than 0.04 or higher than 0.96 using GCOS statistical analysis.

### Comparative analysis epididymal gene expression between two groups

The differentially expressed transcripts after chlorohydrin treatment were defined according to the following criteria: (1) the expression difference between the two groups was two-fold or more, or (2) if the change in *p*-value was below 0.002, then the epididymal transcript expression in the chlorohydrin group increased in comparison to the control group; if the change in *p*-value was higher than 0.998, then the changing trend was considered as decreasing from the chlorohydrin group to the control group.

### Differentially expressed genes classification

The functional analysis of the differentially expressed genes was performed by a GeneSpring GX 7.3.1 (Agilent Technologies Inc. Santa Clara, CA), which is a powerful visualization and analysis solution designed for use with genomic expression data, as it can test simultaneously for Gene Ontology, Kyoto Encyclopedia of Genes, transcription factors (TF) and gene expression in tissues.

### Gene expression validation by Real-Time quantitative polymerase chain reaction (PCR)

The total RNA prepared for the microarray was also used for quantitative RT-PCR. The cDNA samples for RT-PCR analysis were synthesized with oligo-dT primers using the Superscript III First Strand Synthesis System for RT-PCR (Invitrogen, Carlsbad, CA) according to the manufacturer's instructions. The primers used in the PCR reactions are listed in Table. 1. The total volume of 20 μL PCR reactions was prepared by mixing 2.0 μL of cDNA sample, 2.0 μL of 10× PCR buffer, 2.0 μL of 2.5 mM dNTP, 2.0 μL of 5 μM specific gene primer pair, 0.8 μL of 25× SYBR Green I (Fisher Scientific Co., Pittsburgh, PA), 0.2 μL of 5 units/μL Hotstar Taq Polymerase (Qiagen, Valencia, CA) and 11.0 μL of ddH2O. The real-time PCR reactions were performed on a Rotor-Gene 3000 instrument (Corbett Research, Mortlake, Australia) and included an initial incubation at 95°C for 15 min to activate the Hotstar Taq Polymerase. Next, 35 cycles of denaturation (95°C, 15 sec), annealing (58°C, 20 sec) and extension (72°C, 20 sec) were performed with the consecutive acquisition of Sybr fluorescent signals. Finally, the standard curve was established by measuring Beta-actin relative abundance and the quantitation of targeted gene expression was analyzed. Additionally, we also performed routine RT-PCR with agarose gel electrophoresis analysis for the qualitative verification of the results.

**Table 1:** The primers used for Real-Time polymerase chain reaction (PCR).

Target genes	Sense (5'-3')	Antisense (5'-3')	Size of product (bp)
β-actin	CTGGGTATGGAATCCTGT GG	TCATCGTACTCCTGCTTGCTG	290
Gapds	GAATCGCCATTA AAGTCCGT	GGCAAAGTCATCCCAGAG C	141
Lep	TCTGTGGAGTAGAGCGAGGCT	TTCACCCCATTCTGAGTTTGT C	123
Atp6v1g3	TGGGCTTGGAAGGACGAG	GAGACCGACCAGTACAGAATGC	234

## Results

### Effects of ACH treatment on sperm motility

**Figure 1:**
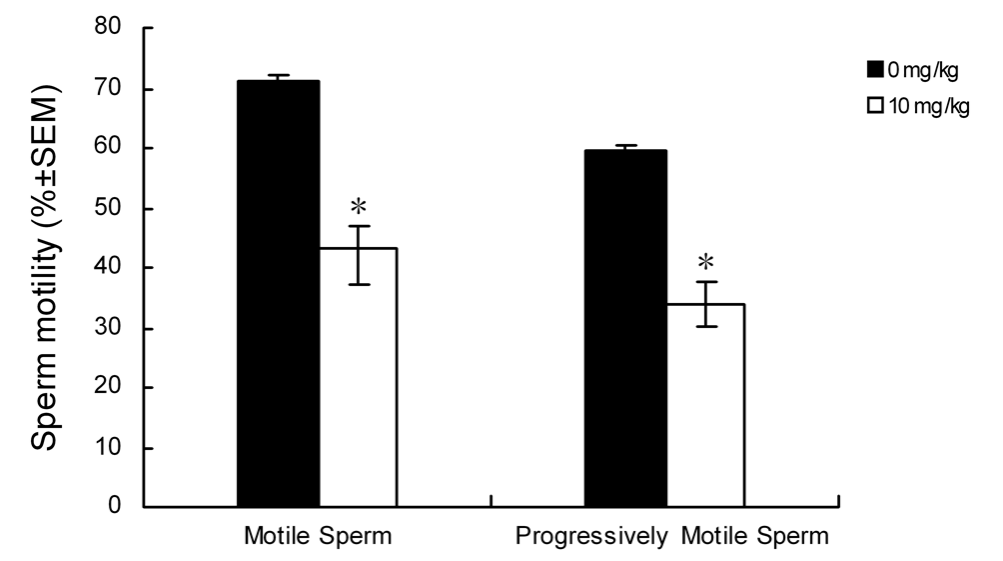
**Effect of ACH treatment on sperm motility (left) and progressive motility (right)**. Data are represented as mean ± S.EM. * *P *< 0.05 versus the control group, n = 8.

The sperm concentration did not significantly decrease in the ACH-treated group as compared to the control group (data not shown). However, there was a significant decrease in the percentage of motile and progressively motile sperm (39% and 45%, respectively) from the cauda epididymis of treated male rats compared with untreated animals (Figure. 1). Furthermore, ACH treatment also caused a significant decrease in other sperm motility parameters, including VSL, VCL, ALH, STR and LIN (Table. 2).

**Table 2:** Effect of ACH treatment on other sperm motion parameters between the two groups (mean ± S.EM.).

ACH	n	VAP (μm/s)	VSL (μm/s)	VCL (μm/s)	ALH (μm)	BCF (Hz)	STR (%)	LIN (%)
0 mg/kg	8	147.5 ± 25.0	107.8 ± 16.4	235.3 ± 49.0	15.1 ± 2.0	25.5 ± 4.8	71.5 ± 3.4	47.0 ± 5.6
10 mg/kg	8	134.6 ± 12.7	89.5 ± 9.7*	194.8 ± 16.6*	12.9 ± 1.6*	26.8 ± 2.1	60.0 ± 6.8*	37.3 ± 4.1*

*P < 0.05 versus the control group. Average path velocity (VAP), Curvilinear velocity (VCL), Straight-line velocity (VSL), Amplitude of lateral head displacement (ALH), Beat cross frequency (BCF), Straightness (STR), Linearity (LIN).

### Effects of ACH treatment on sperm morphology

**Figure 2:**
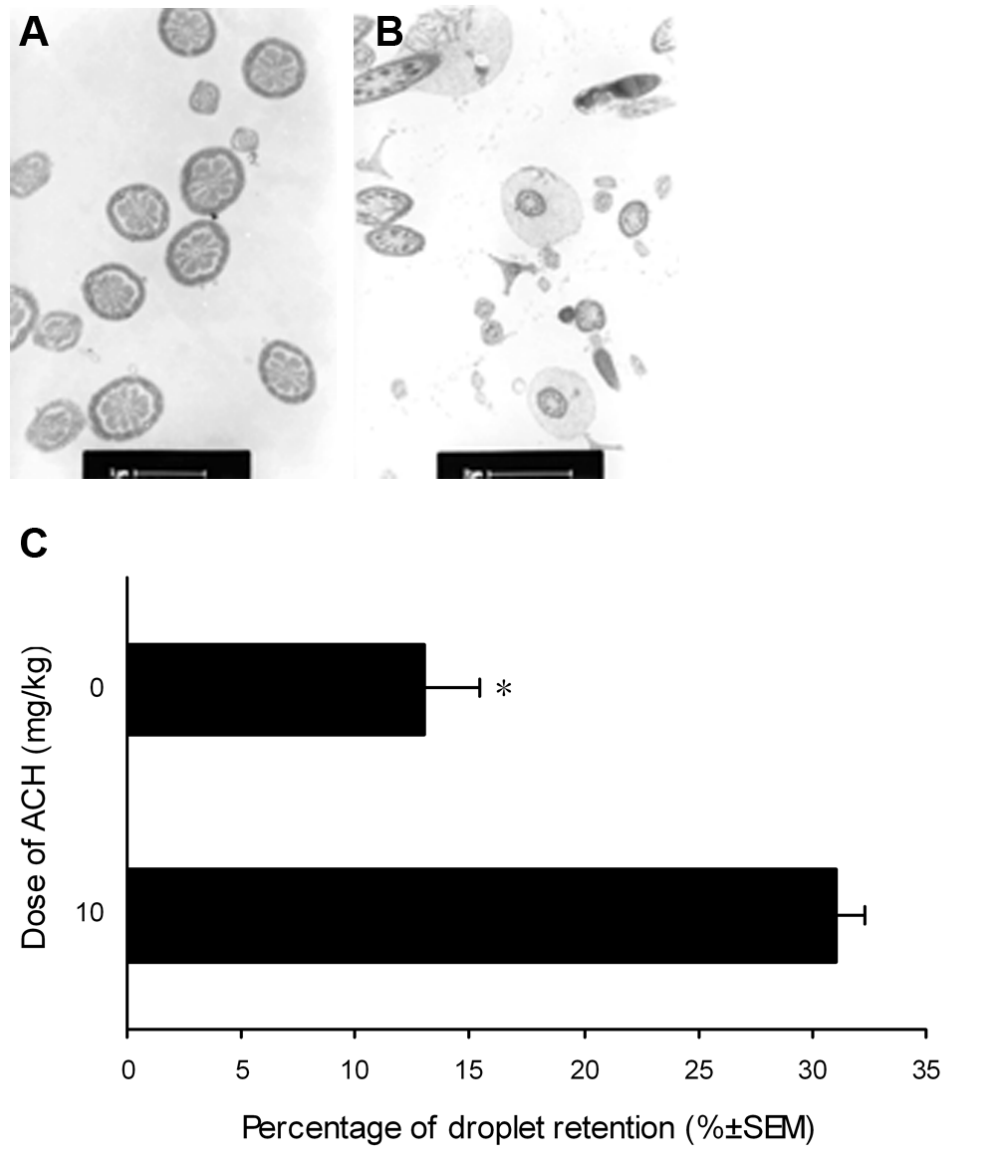
**Electron microscopic analysis of the effect of ACH treatment on sperm morphology. A**: In the control, there are no mid-piece sperm that are surrounded by cytoplasmic droplets; **B**: After ACH treatment, there are some mid-piece sperm surrounded by cytoplasmic droplets (black arrows); **C**: Comparison of the percentage of sperm droplet retention between the control and ACH groups. *P < 0.05 versus the control group.

As expected under phase-contrast microscopy observation, the number of abnormal sperm from the cauda epididymis of treated males was higher than in the controls. The percentage of tailless, headless, broken, angulated and other abnormal sperm all increased significantly with ACH treatment (Table. 3). The sperm maturation process is associated with a series of morphological changes, including the displacement of the cytoplasmic droplet along the mid-piece of sperm travelling from the caput to the cauda epididymal regions [9,22,23]. Analysis of the ultrastructure of sperm from the cauda epididymis revealed a significant increase in the percentage of mid-piece sperm that were surrounded by cytoplasmic droplets after ACH treatment (Figure. 2).

**Table 3:** Comparison between the ACH treated and control groups in the percentage of abnormal cauda epididymis sperm (mean ± S.EM.).

ACH	n	Headless (%)	Tailless (%)	Angulated (%)	Broken (%)	Other (%)	Total (%)
0 mg/kg	8	0.51 ± 0.12	0.83 ± 0.15	3.23 ± 0.29	0.25 ± 0.04	0.26 ± 0.12	5.07 ± 0.83
10 mg/kg	8	4.20 ± 0.32*	5.6 ± 0.89*	6.13 ± 0.46*	1.36 ± 0.32*	0.93 ± 0.42*	18.22 ± 2.56*

*P < 0.05 versus the control group

### Effects of ACH treatment on male mating and fertility

In this study, although all of the males successfully mated with at least one virgin female in proestrus, there was a remarkable difference in the reproduction indices between the control and ACH treatment groups . All of the females that mated with the untreated males were pregnant; in contrast, there was a drastically significant decrease in the pregnancy rate for those females paired with the treated males (Table. 4).

**Table 4:** Effect of ACH treatment on male reproductive indices (%).

ACH	Copulation Index	Pregnancy Index	Fertility Index
0 mg/kg	87.5%(14/16)	100%(14/14)	87.5%(14/16)
10 mg/kg	81.3%(13/16)	14.3%(2/14)*	12.5%(2/16)*

*P < 0.05 versus the control group

### Effects of ACH treatment on reproductive organ weights

There were no significant changes in the body weights between the two groups of rats. As expected, the weight of the testis, a T-dependent organ, was not affected in either group; furthermore, there was no effect of treatment on the weight of the epididymis, seminal vesicle or prostate, all of which are DHT-dependent tissues (data not shown).

### Effects of ACH treatment on Serumal T and DHT

**Figure 3:**
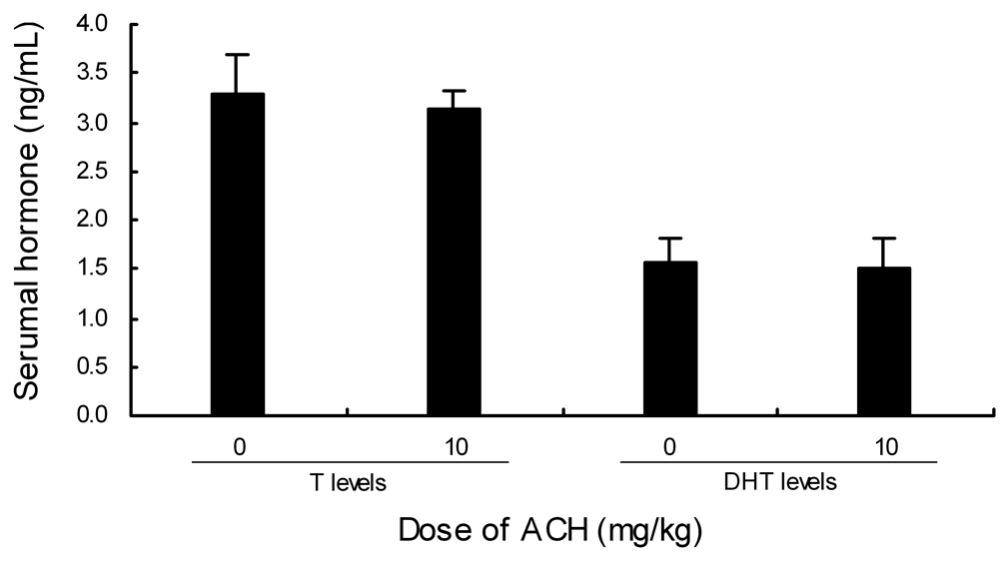
**Effect of ACH treatment on serum T and DHT levels**. Data are represented as mean ± S.EM.

In this study, neither the serum T level nor the serum DHT level was affected with ACH treatment (Figure. 3).

### Effects of ACH treatment on rat epididymal gene expression

**Figure 4:**
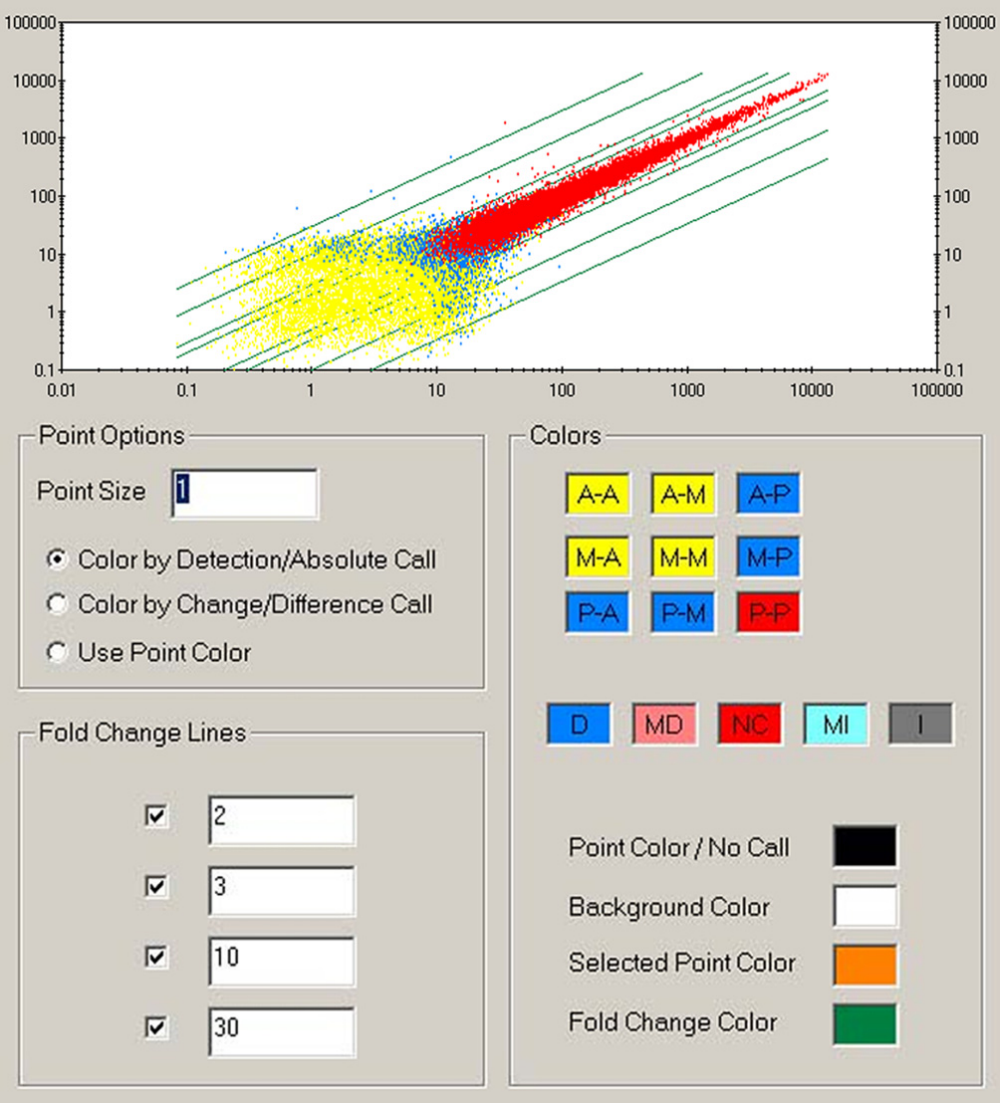
**Scatter plot of epididymal-expressed genes between the two groups**. The yellow points represent genes that are not expressed in either group. The blue points indicate the genes that are expressed in either of the two groups. The red points denote genes that are expressed in both groups. The four upper-left diagonal lines represent the fold of up-regulated gene expression, which is 2, 3, 10 and 30 times, respectively; meanwhile, the four lower-right diagonal lines represent the fold of down-regulated gene expression, which is 1/2, 1/3, 1/10 and 1/30, respectively.

**Figure 5:**
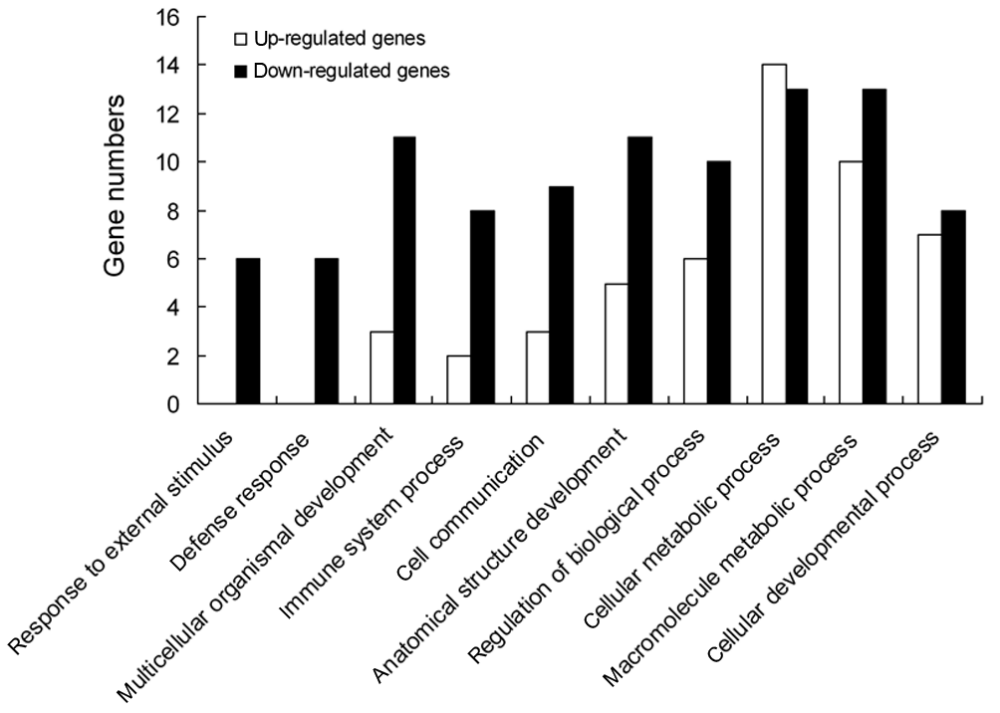
**The functional classification of the differentially expressed genes between the two groups**.

According to the analysis of gene expression along the rat epididymis using GCOS, 17,410 and 17,031 probe sets were detected in the control and ACH treatment groups, respectively, and account for approximately 56.1% and 54.9%, respectively, of the whole genome, which has 31,042 probe sets. When the change in *p*-value was <0.002 or >0.998, there were 90 transcripts with enhanced expression and 79 transcripts with decreased expression in the treatment group as compared to the control group (Figure. 4). Next, we classified the general functions of the down- or up-regulated epididymal genes after chlorohydrin treatment using the GeneSpring gene ontology (GO) analysis (Figure. 5). The genes were involved in macromolecular metabolism and transport, primary metabolism processes, cell metabolism, regulation of biological processes, immunology regulation, hydratase activity and oxidoreductase activity. Among all the differentially expressed genes, we analyzed and screened the down-regulated genes associated with glucose, lipid, protein and other energy metabolism processes, which are considered the major targets of ACH action (Table. 5). Simultaneously, the genes that were up-regulated by chlorohydrin were detected. The genes that negatively regulate sperm maturation and fertility include apoptosis and immune-related genes and have not been reported previously (Table. 6).

**Table 5:** The down-regulated epididymal metabolism-related genes from the ACH treatment group compared with the control group.

Probe set ID	**GenBank NO**.	SLR*	Gene symbol	Gene annotations	Gene ontology
Gapdhs	NM023964	-2.6		glyceraldehyde-3-phosphate dehydrogenase	glyceraldehyde-3-phosphate dehydrogenase activity
1397006_at	BF401586	-5.5	Prkcb1	Protein kinase C, beta 1	protein kinase C activity, ATP binding
1368561_at	NM033352	-1.3	Abcd2	ATP-binding cassette, sub-family D (ALD),	ATPase activity, fatty acid metabolism
1383893_at	AW140864	-1.0	Atp6v1g3	ATPase,	ATP hydrolysis coupled proton transport
1391902_at	AW527377	-2.7	Pgam2	Phosphoglycerate mutase 2	bisphosphoglycerate phosphatase activity
1368547_at	NM130402	-1.5	Ocil	osteoclast inhibitory lectin	sugar binding
1384837_at	AI137672	-1.1	Cd69	CD69 antigen	glucose metabolism, lipid metabolism, JAK-STAT cascade, regulation of cholesterol absorption
1387748_at	NM013076	-1.1	Lep	leptin	glucose metabolism, lipid metabolism, fatty acid catabolism, protein binding, JAK-STAT cascade
1380241_at	AW435376	-3.4	Lrp5	low density lipoprotein receptor-related protein 5	lipid metabolism
1386964_at	NM080775	-1.8	Smgb	neonatal submandibular gland protein B	lipid binding
1374863_at	BI283223	-1.3	RGD1562168	similar to retinoid binding protein 7	lipid binding
1393359_at	AW534487	-2.3	Ap3b2	adaptor-related protein complex 3, beta 2 subunit	intracellular protein transport
1398695_at	BF389056	-5.4	App	Amyloid beta (A4) precursor protein	protein binding
1368642_at	NM031333	-4.4	Cdh2	cadherin 2	protein binding
1387839_at	NM012646	-1.7	Cul2	Cullin 2	protein ubiquitination
1367973_at	NM031530	-1.2	Ccl2	chemokine (C-C motif) ligand 2	G-protein-coupled receptor binding
1370034_at	NP598256	-1.2	Cdc25b	cell division cycle 25 homolog B (S. cerevisiae)	hydrolase activity
1384190_at	BF553848	-1.0	Mapk8ip3	mitogen-activated protein kinase 8 interacting protein 3	serine-type peptidase activity

*The "SLR", which is fully spelt by "Signal log Ratio", indicates the log ratio of signal intensity in ACH treated group in relative to that in control group. The expressions of the genes listed above in ACH-treated group are down-regulated by 2^SLR ^times.

**Table 6:** The up-regulated epididymal genes in the ACH treatment group compared with the control group.

Probe set ID	**GenBank NO**.	SLR*	Gene symbol	Gene annotations	Gene ontology
1368294_at	NM053907^a^	1.4	Dnase1l3	deoxyribonuclease I-like 3	DNA fragmentation during apoptosis
1379794_at	AI029386^a^	1.0	Gzmb	granzyme B	Cytolysis, proteolysis, apoptosis
1387011_at	NM130741^a^	1.0	Lcn2	lipocalin 2	apoptosis
1388202_at	BI395698^b^	3.0	RT1-Aw2	RT1 class Ib, locus Aw2	antigen presentation, MHC class I receptor activity
1370463_x_at	U50449^b^	2.5	RT1-CE16	RT1 class I, CE16	antigen presentation, MHC class I receptor activity
1374342_at	BE096652^b^	1.8	Ly6g6c	lymphocyte antigen 6 complex	immune response
1388203_x_at	BI395698^b^	1.5	RT1-A3	RT1 class I, A3	antigen presentation, endogenous antigen via MHC class I
1385551_at	AW141043^b^	1.3	Mcpc	MHC class I protein complex	MHC class I receptor activity
1398390_at	AA892854^b^	1.0	LOC498335	Small inducible cytokine B13 precursor	immune response

^a ^apoptosis-related genes; ^b ^immune-related genes.

*The "SLR" is fully spelt by "Signal log Ratio", which indicates the log ratio of signal intensity in ACH treated group in relative to that in control group. The expression of the genes listed above in ACH-treated group is up-regulated by 2^SLR ^times.

### RT-PCR analysis of selected gene expression

**Figure 6:**
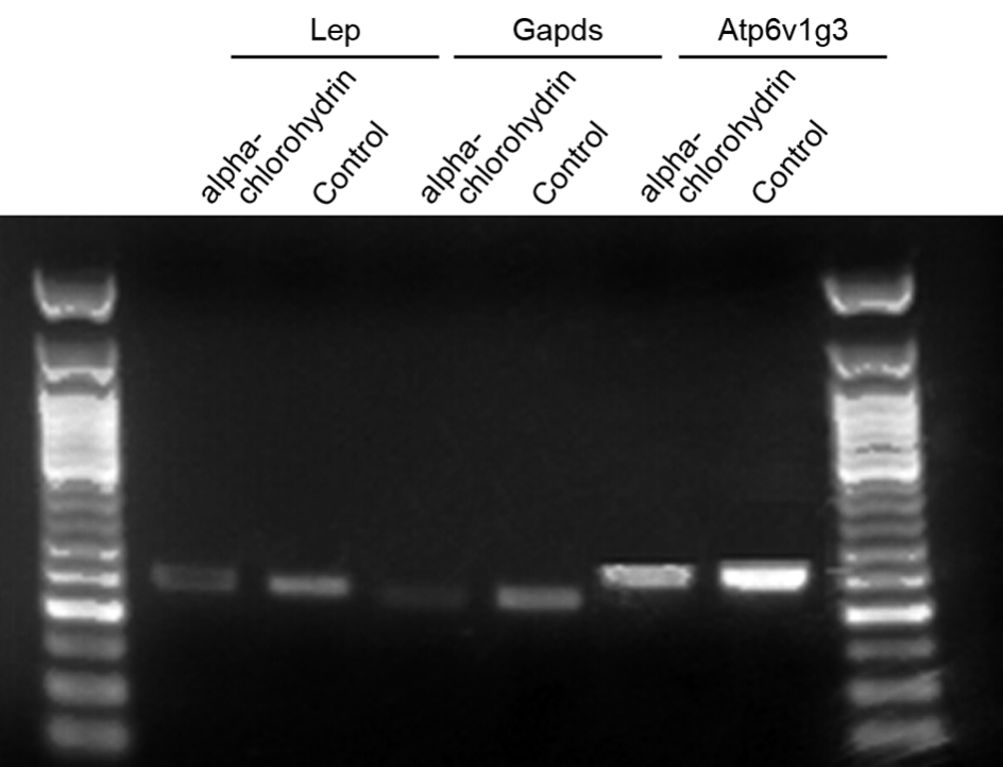
**RT-PCR analysis of selected genes demonstrates the alteration in mRNA expression**.

We performed RT-PCR analysis for several genes including *Gapds*, *Atp6v1g3*, and *Lep *to validate the alteration in gene expression observed by oligonucleotide microarray. The overall results of PCR analysis for these selected genes were consistent with the array data, although the changes in the expression level were not equal to the array results (Figure. 6 and Table. 7).

**Table 7:** Analysis of gene expression data by quantitative PCR.

ACH	Gapds	Lep	Atp6v1g3
0 mg/kg	1.0000	1.0000	1.0000
10 mg/kg	0.3031*	0.4122*	0.3734*

*Significantly different from control at 0.05 level by ANOVA test. Values from the ACH treated group are represented as a ratio relative to the control samples, which have a value of 1.0000.

## Discussion

In the present study, we duplicated a male rat infertility model using ACH administration (10 mg/kg/d, *po*, 10 days) and evaluated changes in sperm motility and morphology, mating index, fertility index and pregnancy index. The results showed that serum androgens remained normal in ACH-treated rats and their sexual abilities weren't negatively affected; thus, it is considered the best method for male contraception. We determined that the down-regulated epididymal genes relate to substance metabolism, which affects epididymal sperm maturation and is presumed to be the major antifertility targets by ACH. Furthermore, we identified and analyzed the epididymal up-regulated genes that are associated with apoptosis and immune function and may be novel sites of action by ACH and other male antifertility agents.

ACH is well known to inhibit the activity of G3PDH, one of the glycolytic enzymes that interferes with epididymal sperm maturation [10,14]. In our study, the transcript of G3PDH was remarkably down-regulated by ACH, and some other enzymes associated with the glycolytic pathway were also inhibited. The results are consistent with previous studies regarding the association between ACH and antifertility. The inhibitory effects of ACH on G3PDH activity *in vitro *are thought to develop via ACH oxidation within the tissues to form 3-chlorolactaldehyde [24]. This metabolite has a compatible chiral conformation to act as an analog of the G3PDH substrate 3-phosphate glyceraldehyde [25]. Inhibition of G3PDH activity induces an ineffective glycolytic cycle by the presence of the saccharide and results in the depletion of ATP. Sperm motion, capacitation and fertility are repressed by ACH due to a deficiency of ATP in the epididymal sperm [26-28].

Our analytic results showed that the expression of some of the ATPase transcripts, such as *Atp6v1g3*, was decreased strikingly. Several previous studies suggested that ATPases were essential for sperm motility [29-31]. The underlying mechanism is potentially that ATPases can facilitate sperm energy production to provide support for sperm material metabolism associated with the maturation process. Our present experiment showed that ACH could inhibit expression of some ATPases in the epididymis, which possibly blocks epididymal energy metabolism and changes epididymal function and material metabolism. Furthermore, when the secretory activity of epididymal epithelial cells is disturbed, the epididymal microenvironment should change subsequently. All of the above alterations lead to dysfunction in the sperm-egg fusion and male fertility by affecting sperm maturation.

From our microarray results, ACH could also significantly inhibit the expression of genes associated with epididymal lipid metabolism, such as *leptin*, *smgb*, and *lrp5*, resulting in changes in the epididymal microenvironment. Among these down-regulated genes, leptin may reduce lipogenesis and enhance lipolysis or energy consumption to control body weight. Further studies found that the epididymides could also synthesize and secret leptin which was considered expressed in spermatozoa and not in epididymis. Leptin is considered an important compound affecting sperm lipid metabolism, protein phosphorylation and glycogen synthetase activity [32-35]. Therefore, leptin may play a role in the process of sperm capacitation through modifying the sperm membrane structure, protein modification and energy storage. Our experimental oligo-microarray and RT-PCR results suggested that ACH could inhibit the expression of leptin in the epididymis, and the metabolism of spermatozoa in the epididymis might be influenced. The inhibition of lipid metabolism increases the retention rates of sperm droplets, which induces abnormal changes in the sperm membrane structure, and the inhibitory effect on protein phosphorylation blocks local protein modification. In addition, the retardation of glycogen synthesis induces a disruption in the energy metabolism of epididymal sperm and is accompanied with abnormal changes regarding sperm motility and morphology. Carbohydrates are essential for the sperm's ability to penetrate through the zona pellucida and for sperm-oocyte binding. If the sperm's storage capacity is attenuated, the sperm-oocyte binding will be dysfunctional, and male fertility will be decreased.

As an alternative nonhormonal method, epididymal immunocontraception has become a hot topic in male contraceptive research and involves many epididymis/testis-specific proteins, such as *Eppin *(epididymal protease inhibitor) and Bin1b [36,37]. Successful immunocontraception would develop an effective, safe, and reversible method for male contraception. In the present study, we found that ACH up-regulated the expression of some epididymal genes associated with the immune reaction, such as *RT1-Aw2*, *Ly6g6c*, and *LOC498335*. These genes/proteins could become potential epididymal specific targets for male immunocontraception and infertility treatment.

## Conclusions

We employed ACH treatment and oligonucleotide microarray analysis to examine the effect of ACH on gene expression in the epididymis. We preliminarily constructed a genome-wide profile of gene expression in the epididymis of rats with ACH-induced infertility, and this analysis potentially provides some new epididymal targets for male contraception and infertility investigations.

## Abbreviations

ACH: alpha-chlorohydrin; G3PDH: glyceraldehyde-3-phosphate dehydrogenase; DHT: dihydrotestosterone; T: testosterone; CASA: computer-assisted sperm analysis; VAP: average path velocity; VCL: curvilinear velocity; VSL: straight-line velocity; ALH: amplitude of lateral head displacement; BCF: beat cross frequency.

## Competing interests

The authors declare that they have no competing interests.

## Authors' contributions

SX participated in all aspects of the study. YZ and YL participated in the design of the study and performed the statistical analysis. LM, YG and JZ carried out the experimental studies. LC conceived of the study, and participated in its design and coordination and helped to draft the manuscript. All authors read and approved the final manuscript.

## References

[B1] Bedford JM Development of the fertilizing ability of spermatozoa in the epididymis of the rabbit J Exp Zool 1966 163 319 329 10.1002/jez.1401630310

[B2] Orgebin-Crist MC Sperm maturation in rabbit epididymis Nature 1967 216 816 818 10.1038/216816a0 6074957

[B3] Blandau RJ Rumery RE The Relationship of Swimming Movements of Epididymal Spermatozoa to Their Fertilizing Capacity Fertil Steril 1964 15 571 579 14236832 10.1016/s0015-0282(16)35401-2

[B4] Jones R Plasma membrane structure and remodelling during sperm maturation in the epididymis J Reprod Fertil Suppl 1998 53 73 84 10645268

[B5] Zhou CX Zhang YL Xiao L Zheng M Leung KM Chan MY Lo PS Tsang LL Wong HY Ho LS Chung YW Chan HC An epididymis-specific beta-defensin is important for the initiation of sperm maturation Nat Cell Biol 2004 6 458 464 10.1038/ncb1127 15122269

[B6] Reyes A Chavarria ME Interference with epididymal physiology as possible site of male contraception Arch Androl 1981 7 159 168 10.3109/01485018108999303 6456706

[B7] Amann RP Johnson L Thompson DL Jr Pickett BW Daily spermatozoal production, epididymal spermatozoal reserves and transit time of spermatozoa through the epididymis of the rhesus monkey Biol Reprod 1976 15 586 592 10.1095/biolreprod15.5.586 826287

[B8] Suzuki K Yu X Chaurand P Araki Y Lareyre JJ Caprioli RM Matusik RJ Orgebin-Crist MC Epididymis-specific promoter-driven gene targeting: a transcription factor which regulates epididymis-specific gene expression Mol Cell Endocrinol 2006 250 184 189 10.1016/j.mce.2005.12.043 16414179

[B9] Henderson NA Robaire B Effects of PNU15 a dual 5alpha-reductase inhibitor, on rat epididymal sperm maturation and fertility Biol Reprod 7706 72 436 443 10.1095/biolreprod.104.033548 15483222

[B10] Jelks KB Miller MG alpha-Chlorohydrin inhibits glyceraldehyde-3-phosphate dehydrogenase in multiple organs as well as in sperm Toxicol Sci 2001 62 115 123 11399799 10.1093/toxsci/62.1.115

[B11] Kakaria VK Sood PP Correlative histochemical and biochemical studies on the adenosine triphosphatase, succinic dehydrogenase and acetylcholinesterase in the epididymis of mice after alpha-chlorohydrin treatment Acta Eur Fertil 1983 14 353 361 6231798

[B12] Nag S Ghosh JJ Epididymal and testicular enzymes as monitors for assessment of male antifertility drugs J Steroid Biochem 1979 11 681 688 10.1016/0022-4731(79)90100-6 491633

[B13] Sood PP Majid MA Qualitative and quantitative changes of acid and alkaline phosphatases in the testis and epididymis of mice in relation to single high dose of alpha-chlorohydrin Acta Eur Fertil 1987 18 33 38 3630567

[B14] Kalla NR Kaur S Ujwal N Mehta U Joos H Frick J alpha-Glucosidase activity in the rat epididymis under different physiological conditions Int J Androl 1997 20 92 95 10.1046/j.1365-2605.1997.t01-1-00039.x 9292319

[B15] Crabo BG Zimmerman KJ Hunter AG Graham EF Moore R Effect of alpha-chlorohydrin on epididymal sperm and epididymal plasma in swine Arch Androl 1979 3 79 87 10.3109/01485017908985052 485662

[B16] Caflisch CR DuBose TD Jr Effect of alpha-chlorohydrin on in situ pH in rat testis and epididymis Contraception 1990 41 207 212 10.1016/0010-7824(90)90149-P 2311406

[B17] Tsang AY Lee WM Wong PY Effects of antifertility drugs on epididymal protein secretion, acquisition of sperm surface proteins and fertility in male rats Int J Androl 1981 4 703 712 10.1111/j.1365-2605.1981.tb00754.x 7319653

[B18] Hinton BT Hernandez H Howards SS The male antifertility agents alpha chlorohydrin, 5-thio-D-glucose, and 6-chloro-6-deoxy-D-glucose interfere with sugar transport across the epithelium of the rat caput epididymidis J Androl 1983 4 216 221 6874562 10.1002/j.1939-4640.1983.tb00758.x

[B19] Gill SK Guraya SS Effects of low doses of alpha chlorohydrin on the lipid metabolism of the rat testis and epididymis--a correlative histochemical and biochemical study Int J Fertil 1983 28 43 48 6134687

[B20] Lye RJ Hinton BT Technologies for the study of epididymal-specific genes Mol Cell Endocrinol 2004 216 23 30 10.1016/j.mce.2003.10.072 15109741

[B21] Fox SA Yang L Hinton BT Identifying putative contraceptive targets by dissecting signal transduction networks in the epididymis using an in vivo electroporation (electrotransfer) approach Mol Cell Endocrinol 2006 250 196 200 10.1016/j.mce.2005.12.045 16423449

[B22] Fawcett DW Phillips DM Observations on the release of spermatozoa and on changes in the head during the passage through the epididymis J Reprod Fertil 1969 6 405 418

[B23] Hermo L Dworkin J Oko R Role of epithelial clear cells of the rat epididymis in the disposal of the contents of cytoplasmic droplets detached from spermatozoa Am J Anat 1988 183 107 124 10.1002/aja.1001830202 2849296

[B24] Cooney SJ Jones AR Inhibitory effects of (S)-3-chlorolactaldehyde on the metabolic activity of boar spermatozoa in vitro J Reprod Fertil 1988 82 309 317 3339589 10.1530/jrf.0.0820309

[B25] Stevenson D Jones AR Production of (S)-3-chlorolactaldehyde from (S)-alpha-chlorohydrin by boar spermatozoa and the inhibition of glyceraldehyde 3-phosphate dehydrogenase in vitro J Reprod Fertil 1985 74 157 165 4020765 10.1530/jrf.0.0740157

[B26] Dravland E Meizel S Stimulation of hamster sperm capacitation and acrosome reaction *in vitro *by glucose and lactate and inhibition by the glycolytic inhibitor alpha-chlorhydrin Gamete Res 1981 4 515 523 10.1002/mrd.1120040605

[B27] Toth GP Wang SR McCarthy H Tocco DR Smith MK Effects of three male reproductive toxicants on rat cauda epididymal sperm motion Reprod Toxicol 1992 6 507 515 10.1016/0890-6238(92)90035-R 1288760

[B28] Slott VL Jeffay SC Suarez JD Barbee RR Perreault SD Synchronous assessment of sperm motility and fertilizing ability in the hamster following treatment with alpha-chlorohydrin J Androl 1995 16 523 535 8867601

[B29] Ghoshal S Sengupta T Dundung SR Majumder GC Sen PC Characterization of a low-molecular-mass stimulator protein of Mg2+-independent Ca2+-ATPase: effect on phosphorylation/dephosphorylation, calcium transport and sperm-cell motility Biosci Rep 2008 28 61 71 10.1042/BSR20070016 18241199

[B30] Sanchez G Nguyen AN Timmerberg B Tash JS Blanco G The Na, K-ATPase alpha4 isoform from humans has distinct enzymatic properties and is important for sperm motility Mol Hum Reprod 2006 12 565 576 10.1093/molehr/gal062 16861705

[B31] Withers S Cartwright EJ Neyses L Sperm phenotype of mice carrying a gene deletion for the plasma membrane calcium/calmodulin dependent ATPase 4 Mol Cell Endocrinol 2006 250 93 97 10.1016/j.mce.2005.12.028 16442703

[B32] Nazian SJ Leptin secretion from the epididymal fat pad is increased by the sexual maturation of the male rat J Androl 2001 22 491 496 11330650

[B33] Aquila S Gentile M Middea E Catalano S Morelli C Pezzi V Ando S Leptin secretion by human ejaculated spermatozoa J Clin Endocrinol Metab 2005 90 4753 4761 10.1210/jc.2004-2233 15944217

[B34] Jope T Lammert A Kratzsch J Paasch U Glander HJ Leptin and leptin receptor in human seminal plasma and in human spermatozoa Int J Androl 2003 26 335 341 10.1111/j.1365-2605.2003.00434.x 14636218

[B35] Ando S Aquila S Arguments raised by the recent discovery that insulin and leptin are expressed in and secreted by human ejaculated spermatozoa Mol Cell Endocrinol 2005 245 1 6 10.1016/j.mce.2005.09.011 16274924

[B36] O'Rand MG Widgren EE Sivashanmugam P Richardson RT Hall SH French FS VandeVoort CA Ramachandra SG Ramesh V Jagannadha Rao A Reversible immunocontraception in male monkeys immunized with eppin Science 2004 306 1189 1190 10.1126/science.1099743 15539605

[B37] Li P Chan HC He B So SC Chung YW Shang Q Zhang YD Zhang YL An antimicrobial peptide gene found in the male reproductive system of rats Science 2001 291 1783 1785 10.1126/science.1056545 11230693

